# The Enriching Interplay between Openness and Interest: A Theoretical Elaboration of the OFCI Model and a First Empirical Test

**DOI:** 10.3390/jintelligence6030035

**Published:** 2018-08-01

**Authors:** Matthias Ziegler, Titus A. Schroeter, Oliver Lüdtke, Lena Roemer

**Affiliations:** 1 Institut für Psychologie, Humboldt-Universität zu Berlin, Rudower Chaussee 18, 12489 Berlin, Germany; titus.schroeter@posteo.de (T.A.S.); roemerle@hu-berlin.de (L.R.); 2 Leibniz Institute for Science and Mathematics Education, Kiel University, Olshausenstraße 62, D-24118 Kiel, Germany; oluedtke@ipn.uni-kiel.de; 3 Centre for International Student Assessment, Kiel University, Olshausenstraße 62, D-24118 Kiel, Germany

**Keywords:** crystallized intelligence, knowledge, Openness to Experience, interests, Investment Theory, OFCI model, TESSERA

## Abstract

The Openness-Fluid-Crystallized-Intelligence (OFCI) model posits long-term relations between Openness and cognitive abilities and has been successfully tested with longitudinal data. However, research on the developmental interplay between cognitive abilities and personality exists only sparsely. The current paper focuses on a theoretical development of the OFCI model which suggests micro-level mechanisms underlying the long-term development. Specifically, within-situation relations between Openness, interests, situational perception, cognitive abilities, and emotions are proposed to explain longitudinal relations between Openness and cognitive abilities. Using experience sampling, selected parts of this elaboration were empirically scrutinized in a first test of the proposed ideas. Openness and specific interest both varied substantially across situations and covaried systematically. In interaction with an indicator of fluid intelligence, this covariation was related to an indicator of crystallized intelligence. The paper contributes to theorizing the intertwined development of personality and cognitive abilities, and highlights the importance of within-situation research for explaining long-term development.

## 1. Introduction

In recent years there has been an increased interest into the developmental paths of important human traits. For example, many studies have focused on the development of cognitive abilities, e.g., [1], also exploring potential interactions between them [2]. At the same time, research on the development of personality traits such as the Big Five has also been quite successful [3,4]. However, research combining both research streams remains sparse [5,6]. This is even more startling when considering the existence of models proposing intertwined developmental trajectories for fluid intelligence, crystallized intelligence, interests, and the personality trait of Openness to experience [7,8,9,10,11,12]. One of the more recent models, the Openness-Fluid-Crystallized-Intelligence (OFCI) model [12], has been successfully tested for young and late adulthood [11] using longitudinal data. One reason why the existence of such models might have failed to spark more research into the developmental interplay between cognitive abilities and personality might be the lack of concrete mechanisms as explanations for the proposed relations. The current paper aims at contributing to closing this gap. We will do so by first elaborating the OFCI model with regard to the interplay between Openness and interests, specifically considered from a micro-level perspective. Second, we will report a first test of these ideas using data from an experience sampling study.

### 1.1. The OFCI Model

**Figure 1:**
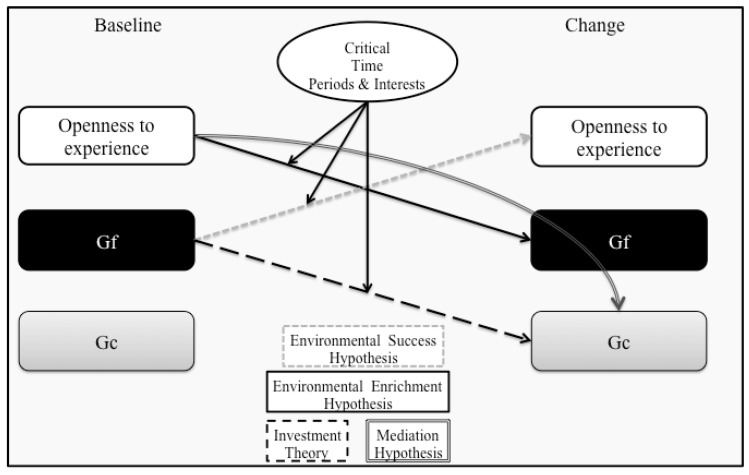
The Openness-Fluid-Crystallized-Intelligence (OFCI) model according to Ziegler, Danay, Heene, Asendorpf and Bühner [12].

The OFCI model is a developmental process model integrating Openness, fluid intelligence (Gf), and crystallized intelligence (Gc) [11,12]. Specifically, it describes four different mechanisms (see Figure 1): First, based on the environmental enrichment hypothesis it is argued that Openness leads people into more enriched environments which helps them to practice and thereby positively influence the development of their fluid intelligence. Second, there is also a reversed impact of Gf on the development of Openness, which is based on the environmental success hypothesis, also see [13]. According to this hypothesis, individuals with higher levels of Gf have a higher probability of successfully mastering challenging new tasks which influences how such situations are perceived and whether they will be actively pursued in the future; ultimately, these processes manifest in altered levels of Openness [14]. Third, the model assumes that Openness has an effect on Gc, which is mediated by its impact on Gf (mediation hypothesis). This hypothesis assumes that individuals higher in Openness are more likely to encounter new situations; however, simply experiencing such situations might not be sufficient to accumulate Gc. Instead, employing fluid abilities to make sense of the situation is seen as a necessary prerequisite. Fourth, the OFCI model also builds on the investment theory by Cattell [14], which proposes an impact of Gf on the development of Gc. The processes outlined above are meant to occur only within critical time frames, when open behavior can lead to new and enriching environmental influences. This is based on the ideas delineated in the trait activation theory by Tett and Burnett [15]. The general idea here is that interindividual differences in a specific trait are more likely to manifest if people perceive situational cues calling for behavior related to the trait [16]. With regard to the OFCI model this means that differences in open behavior are more likely to occur if the current state of living allows or even calls for such behavior.

The environmental enrichment path within the OFCI model seems especially important, as it is the starting point from which the personality trait Openness influences Gc. The theoretical assumptions underlying this path have been outlined rather abstractly before. In the original OFCI model it was also proposed that interests affect this path. This was based on ideas by Cattell [14] and Ackerman [9]. Yet, the specific mechanisms describing the interplay between Openness and interests affecting ability were packed into a black box. Here, we will try to shed light into this black box by proposing and empirically testing a mechanism linking Openness and interest.

**Environmental enrichment based on Openness and interest.** Openness to experience is a rather heterogeneous trait, which at the broadest level can be split into the two aspects: Openness and Intellect [17]. Roughly, the Openness aspect is related to being receptive for aesthetics, emotions, and sensory information. With regard to engagement, Openness has been shown to predict affective engagement [18]. The Intellect aspect on the other hand is characterized by cognitive engagement [18]. With regard to learning, a recent study by von Stumm [19] attested more relevance to Openness than to intellect. Clearly, more research with regard to the two aspects and learning is needed. In the context of environmental enrichment, the facets that are subsumed within the Intellect aspect seem to be of particular importance, even though the work by von Stumm suggests Openness cannot be neglected. Recent studies have shown that Intellect facets foster the acquisition of knowledge and Gc, e.g., [16,20]. Further, the aspect of Intellect, and the facets Ideas and Action were shown to be related to fluid intelligence, e.g., [12,21,22,23]. This empirically underscored relation between the Intellect aspect and intelligence is in line with the integrative framework of Intellect [24]. In this framework, Mussel [24] differentiated between a motivational process component and an operational component of Intellect. The motivational component consists of the two processes: Seek and Conquer. Whereas the Seek process leads individuals into new and stimulating situations where learning opportunities can be experienced, the Conquer process is thought to take place once these situations are encountered, leading people to actually learn. Within the framework, the motivational processes are crossed with operational components. The three operations—Think, Learn, and Create—map onto cognitive abilities (e.g., Think mirrors fluid intelligence; see [24]), nevertheless the process component seems to constitute a more straightforward frame for explaining the relationship between Openness and cognitive abilities. Within the OFCI model the Seek process can be considered as the starting point for environmental enrichment. In that sense we consider Openness as the general willingness to seek out and engage with new and stimulating situations/information. Consequently, people higher in Openness, especially in the Intellect facets, should be more willing to seek and conquer new stimuli, thereby enriching their environment and ultimately gathering Gc. Based on these empirical and theoretical underpinnings we will focus the Intellect facets here.

At this point it is important to point out once more that this process is thought to be influenced by interests. More specifically, interests are thought to steer the direction in which the energy provided by Openness is directed. The general idea is that the interests define the field in which one seeks and conquers new information and thus gathers Gc [12,25]. Taking this idea one step further and integrating it into the OFCI model’s environmental enrichment part would mean that while Openness is the general willingness to seek and engage with new information, a match between the type of information and a person’s interest should further energize the enrichment process by increasing and prolonging the willingness to engage. In other words, the combination of high Openness and high interest matching the content within a learning situation should be especially advantageous for the ensuing processes within the OFCI model and therefore, ultimately for the development of Gc. Before further elaborating this idea, we will look at prior research focusing relations between interests and the other constructs within the OFCI model.

### 1.2. The OFCI Model and Interests

The RIASEC model by Holland [26] most likely is the most widely used theory of (vocational) interests. Within this model the interest types realistic (R), investigative (I), artistic (A), social (S), enterprising (E), and conventional (C) are postulated. Moreover, it is assumed that each interest comprises a set of specific activities representative of the respective interest field. This model was applied in many studies and therefore much is known about the relation with the constructs within the OFCI model.

**Openness and interests.** Mount, et al. [27] published a meta-analysis investigating the relations between the Big Five traits and the RIASEC interests. For Openness the largest correlations were reported with investigative (*ρ* = 0.25) and artistic (*ρ* = 0.42) interests. While this seems to imply that especially artistic interests are related to Openness, one has to consider the faceted nature of Openness. In fact, Sullivan and Hansen [28] could show that the relation between Openness and artistic interests can largely be accounted for by the Aesthetics facet of Openness. The facets more representative of the Intellect aspect are rather related to investigative interests. This highlights the importance of differentiating the two Openness aspects as well as interests when considering their role in environmental enrichment and specifically the interplay of these constructs. This idea of specific relations between Openness facets and interests has been verified by Wille et al. [29]. Those authors reported that high investigative interest (i.e., a preference for abstract thinking and a need to understand theories) is related to the Openness facet Ideas, which can be subsumed under the Intellect aspect [17]. Within the OFCI model this would mean that Openness is likely to generally covary with investigative and artistic interests, and that knowledge acquisition benefits especially from the aforementioned match between content and interest. In other words, a high interest does not further energize environmental enrichment if it does not match the content of the new information. That is, an interplay between Openness and investigative interests should be especially beneficial for the acquisition of knowledge that is rather abstract and theory laden. In the current pilot study, the data collected and used later were from psychology students, therefore, the match between content and corresponding interest will be further clarified regarding the content of a psychology major. A psychology degree program typically includes many subjects that transport complex theories. Thus, within this context investigative interests should match the new information’s content and consequently be of importance for knowledge acquisition.

Despite their general relationship with Openness, artistic interests should be less relevant for the acquisition of abstract knowledge within a psychology major. Artistic interests are more reflective of creative processes, thus, no impact on the acquisition of knowledge within a psychology degree program is expected. Importantly, this is not saying that artistic interests do not contribute to environmental enrichment and Gc development. On the contrary, this is a very likely process. However, we want to stress again the importance of the match between interest and the content of the new information—which is not expected to occur for artistic interests within a psychology major.

Taking a closer look at the other interests, one interest, which might influence learning in the field of psychology, could be social interests. As Wille et al. [29] argued this interest is related to activities where others are being helped in a specific way. Pozzebon et al. [30] could show that this interest type is elevated for majors like psychology. While this does not directly support the idea of a beneficial effect on learning, it seems necessary to also include this interest type here.

Summing up, there is research pointing out the importance of interests for learning in specific fields. For the field of psychology targeted in this study investigative and social interests were reported as relevant. Specifically, investigative interests are related to the Intellect facets of Openness supporting the hypothesis that a high level of both in a given situation should positively affect learning and the development of Gc when it comes to abstract and theory laden content. However, due to the relations between Openness and artistic as well as social interests, (also see [31], for findings showing reciprocal developmental effects) it seems necessary to include these interests types as control variables.

**Interests, Gf, and Gc.** Ackerman and Heggestad [32] published a meta-analysis estimating construct correlations between different abilities and personality traits as well as interests, also see [22,33]. In general, small to moderate correlations were reported. More specifically, investigative and realistic interests were seen as more related to mathematical or numerical abilities. Literacy or verbal ability was more related to investigative and artistic interests. Again, investigative interests stand out by being related to both ability areas. Interestingly, both ability domains were found to influence success in the early stages of a psychology program [20].

Taking a more nuanced approach, which is more in line with the OFCI model and focuses on Gf and Gc, Ackerman [34] could show that both investigative and artistic interests can contribute to the development of Gc. Thus, to sum up, there are small to moderate relations between interests and cognitive abilities. As before, specifically investigative and artistic interests are considered to be influential for the development of Gc.

### 1.3. An Elaboration of the OFCI Model

The ideas brought forward above can be understood as an elaboration of the OFCI model, in particular the role of interests within the environmental enrichment part. To summarize, Openness, specifically the Intellect related facets, is considered as the general willingness to seek and engage with new information. If this information matches the interest of a person this will further energize the engagement with the new information. Consequently, high Openness and high information-matching interest within a learning situation will positively affect learning and thereby the development of the cognitive abilities Gf and Gc.

Of further importance here is the emphasis on the situation. So far the OFCI model only incorporated a trait level perspective in terms of constructs and a macro-level process perspective in terms of effects. The elaboration proposed here also means to include a state level perspective for constructs and a micro-level perspective in terms of effects.

**Taking on a state level perspective.** The suggested elaboration of the OFCI model is based on acknowledging the importance of intraindividual variability in stable traits. Personality traits are conceived as relatively durable patterns of interindividual differences in behavior, feelings, and thoughts [35]. A large body of research supports the utility of personality traits to predict future behavior [36]. However, in specific situations, an individual’s behavior might deviate from these general patterns: momentary behavior fluctuates substantially across situations [37]. Put differently, personality states, defined as momentary behavioral expressions of the corresponding trait [38], can deviate from corresponding personality traits. These apparently conflicting views on personality—variability versus stability—were reconciled in the Whole Trait Theory [38]. According to this model, personality traits are best understood as density curves of momentary states. State expressions of personality vary due to situational factors around a mean, which corresponds to one’s trait level. Hence, when examining concrete behavior, one should take the state level into account [4,39]. Applying this line of thought on the OFCI model, it is not assumed that high trait levels of Openness and matching interest are beneficial per se. Rather than that, the within-situation manifestations of both constructs—that is, their states—should be examined.

While there is abundant literature on state fluctuations of personality [40], research on intrapersonal variability of vocational interests is rarer. The fluctuating nature of interests has been documented in the field of educational psychology, however. Their focus on so-called situational interests shows large amounts of intrapersonal variability of momentary experiences of interest [41]. Thus, it seems likely that interests as examined here display variations in within-situation manifestations.

**Long-term development via short-term processes.** Measures of real-time expressions of personality characteristics yield insights into the micro-level relations between the constructs of interest. The idea of long-term personality development based on short-term processes has recently been formalized by Wrzus and Roberts [4] within their TESSERA framework. There it is assumed that on a micro-level, sequences of triggering situations (T), expectancies (E), states and state expressions (SS), and reactions (RA) occur, which lead to development of the respective traits on a macro-level. Within the environmental enrichment path of the OFCI model this would mean that learning situations or the occurrence of new information act as triggers for such a sequence. Specific expectancies as to how to behave in such situations might be one aspect important in switching the manifestation of a trait on or off. For example, being in a student role and encountering new information within a lecture might activate investigative interests and Openness resulting in higher state expressions for these traits. Higher trait levels are likely to be related to higher state levels underscoring the relevance of considering the general trait levels as well. Importantly, we assume that this process is influenced by situational perception [42,43]. In particular we propose that perceiving a situation in a specific way will make manifestations of Openness and interests more likely [44]. Based on the situational taxonomies DIAMONDS [42] and Situation 5 [45,46] we hypothesize that situational perceptions such as positivity or negativity, cognitive load, duty, and intellect can act as facilitators or blockers [15,16] of behavioral manifestations of Openness and interests.

**Figure 2:**
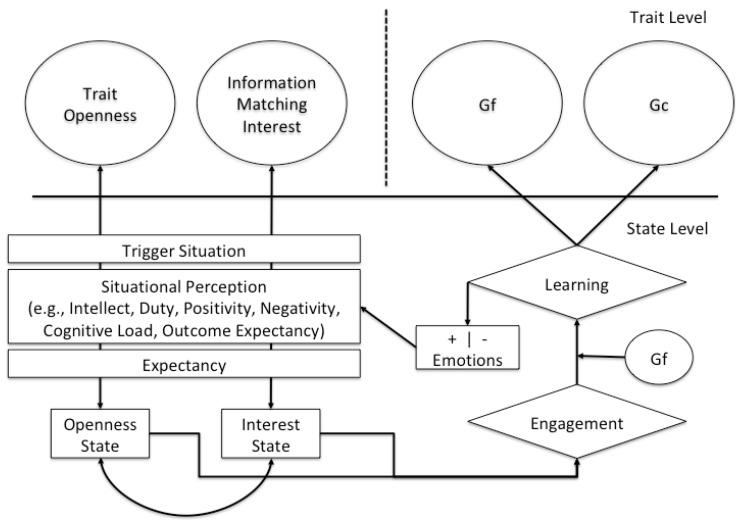
Adaptation of the TESSERA model [4] to the environmental enrichment path of the OFCI model. The dotted line reflects a difference in time. Manifest variables are depicted by rectangles and latent variables by circles.

The reaction within the described sequence could be the willingness to conquer the new material and thus, the engagement with it. Clearly, Gf will moderate the success in actually doing so. It is reasonable to assume that engagement alone does not suffice to learn. Understanding of new material is likely to be moderated by fluid abilities. As a consequence, this is also where the training of fluid abilities occurs. They are needed to conquer the new information. Importantly, learning will be associated with positive (or negative) emotions [47]. This strengthens (or weakens) the relation between both traits (i.e., Openness and interest) thereby influencing the likelihood of their future co-occurrence within similar situations. Assuming learning success and positive emotions, this TESSERA sequence becomes reinforced [4] and thus, more likely (as symbolized by the double-headed errors within Figure 2) which would lead to environmental enrichment and ultimately the positive development of Gf and Gc.

Additionally, Wrzus and Roberts [4] point out further necessary conditions for the TESSERA sequences to induce long-term change. Some of these relate to required discrepancies between expressed states and one’s typical trait level, or to incongruences between reactions and one’s personality. In the current research long-term development focuses on a positive development of knowledge. Therefore, the necessary discrepancies are given when taking the novelty of the content into account: higher state expressions of Openness and interests triggered by content that was formerly unknown can be understood as differing from the typical state expressions towards this content (which, due to its novelty, did not exist before) or known content (even from the same knowledge domain). This relationship between novelty and interest was scrutinized by Fayn et al. [48], who presented evidence that the two constructs were more strongly related for people scoring high on Openness. Here it has to be kept in mind that this relation differs for the aspects of Openness and Intellect [18], with Openness predicting affective and Intellect predicting cognitive engagement.

### 1.4. Aims of the Current Pilot Study

The current pilot study was conducted in order to test some selected aspects of the elaborations presented above. Considering the resources needed to conduct a full-scale test of the complete model we will focus on the micro-level processes, specifically the assumed interplay between Openness and interests. We will also focus on Intellect and interest states, with all state measures being collected using experience sampling. Situational perceptions will not be considered at this point, we do, however, include proxies for Gf and knowledge in a more longitudinal part of the design. Thus, this study should be understood as a first step towards testing the full model proposed here.

To test some of the assumed ideas, different hypotheses will be tested. First, we will test whether there is sufficient intraindividual variability within Openness and interests (Hypothesis 1). Sufficient in this context means to achieve ICCs that are comparable in size to the ones reported in previous studies on personality traits [40]. This is a necessary prerequisite for the idea that Openness and interest states are of importance at all. Second, we will test whether these states covary within situations (Hypothesis 2). Finally, we will test the idea that a generally high covariation between Openness and interest states can be understood as a willingness to engage, which interacts with Gf to foster learning and the acquisition of knowledge (Hypothesis 3). As outlined above, we will focus on the Intellect aspect of Openness, investigative, artistic, and social interests. Here it is assumed that investigative interests should be the relevant energizer along with Intellect. Considering the breadth of this interest type, we also created a more specific measure of investigative interest into psychology.

## 2. Methods

### 2.1. Sample

The sample consisted of *N* = 54 (51 female) undergraduate psychology students enrolled at a German university. The average age was 23.28 (*SD* = 5.39); 39 students were in their first to second semester, 15 in higher semesters. Recruitment was conducted via an e-mail distribution list for psychology students, a popular online social network, and oral reminders in the context of lectures at the institute.

### 2.2. Procedure

Data collection was conducted in three steps: (1) Respondents filled out an online-questionnaire and tests were administered in a computer laboratory; (2) For the experience sampling component participants received tablet computers and filled out a questionnaire with items presented in random order by a custom-designed application. The questions were answered three times a day (prompted by an alarm at 11 a.m., 3 p.m., and 7 p.m.) over the course of three weeks. 92% of the sample followed this routine. However, some participants left out some ratings and had to follow the procedure for a longer time (on average 22.6 days to obtain all ratings); (3) Finally, results for first-semester exams were collected from the examination office at the end of the semester, approximately three months after data collection. As incentives, participants earned course credit and were allowed to use the tablet computers to their liking during the experience sampling period.

### 2.3. Measures

**Pre-test measures.** Openness was assessed by a German Big Five questionnaire [49] based on items from the International Personality Item Pool (IPIP) [50]. It consists of 210 items assessing seven to nine facets for each of the five domains (Neuroticism, Extraversion, Openness to Experience, Agreeableness, and Conscientiousness) with five items per facet. Participants are asked to rate statements about themselves with respect to typical behaviors or reactions on a five-point Likert-type scale, ranging from 1 *strongly disagree* to 5 *strongly agree*. Regarding Openness, the used personality questionnaire does not differentiate the two aspects Intellect and Openness but strongly focuses on the Intellect related facets. Thus, for this study, ratings were aggregated on the trait level only. The internal consistency for Openness (comprising 45 items) was Cronbach’s *α* = 0.87.

For subject-specific interest (interest in psychology) participants were asked to rate the statement “I am very interested in …” concerning nine sub-disciplines of psychology (e.g., “research methods” or “clinical psychology and psychotherapy”) on a five-point Likert-type scale, ranging from 1 *strongly disagree* to 5 *strongly agree*. These ratings were aggregated as an index of interest in psychology with an internal consistency of *α* = 0.57.

Regarding domain-specific interests, participants filled out the General Interest Structure Test–revised (“Allgemeiner Interessen-Struktur-Test–Revision”, AIST-R; [51]), a German inventory of interests according to Holland’s [26,52] RIASEC model. It consists of 60 items, with 10 for each of the dimensions: realistic, investigative, artistic, social, enterprising, and conventional. Participants are asked to rate their interest towards certain occupational activities on a five-point Likert-type scale ranging from 1 *I am not interested in this at all; I do not like doing this* to 5 *I am very interested in this*; *I like doing this very much*. Internal consistency was *α* = 0.86 for investigative, 0.89 for artistic, and 0.84 for social interest.

As part of the laboratory assessment, participants were asked to complete Raven’s Advanced Progressive Matrices (RAPM) [53]. RAPM were designed to measure (nonverbal) reasoning ability in adults and adolescents of above-average intelligence. Participants are requested to solve as many as possible out of 48 increasingly difficult matrices problems within 40 min. In this study participants were asked to solve as many as possible from a subset of 15 matrices, followed by two other cognitive tasks measuring reaction times (all on the computer) within 30–40 min in total. The reaction time tasks were not of interest here. The ratio of correct solutions (with a range of 0 to 1) was used as an indicator of Gf (Cronbach’s *α* = 0.78).

**Experience sampling measures.** The experience sampling questionnaire comprised 25 constructs including the Big Five, Holland’s RIASEC interests, interest in psychology, and 13 others that were not used in the present study. Due to this breadth only one item was used to assess each construct to avoid overburdening participants. For the present study prototypical single-item measures for present state Openness (especially the Intellect aspect), subject-specific interest (interest in psychology), and domain-specific interests (investigative, artistic, and social interest) were included in a block following the instruction to answer the statements according to the way the respondent felt right at the moment. These were to be answered using a Likert-type scale with four levels labeled *strongly disagree*, *disagree*, *agree*, and *strongly agree*. Openness was assessed using the statement “At the moment I am enthusiastic, learning something new”. The item loading was 0.4. Interest in psychology was assessed using the statement “At the moment I am interested in psychology”. Investigative interest was assessed using the statement “At the moment I would be interested to occupy myself with things unexplored” (item loading was 0.71). Artistic interest was assessed using the statement “At the moment I would be interested to design something from an artistic point of view” (item loading was 0.72). Social interest was assessed using the statement “At the moment I would be interested to take care of needy children or adults” (item loading was 0.76). The items assessing Openness, as well as investigative, artistic, and social interest were selected from the above mentioned questionnaires: from the three items with the highest factor loadings, the item best-representing the respective construct was chosen by expert judgment (and prefixed by “At the moment”).

**Post-test measure.** Performance results for the four first-semester examinations were aggregated (grade point average, GPA) as an indicator of knowledge, which is a domain-specific facet of Gc [54]. The sample contained 15 students from higher semesters. Those students were also included in the analyses. However, this also means that the grades for those students were given before data collection, which implies that causal interpretations are not possible. The four first-semester examinations included an exam for (1) “research methods and academic research and writing”—covering contents from a lecture and two tutorials—and exams on (2) “biological psychology”, (3) “learning and memory”, and (4) “thinking and theoretical foundations of general psychology”—the latter three being based on one lecture each. Grades ranged from 1 to 4 with lower values indicating better performance. The exams are a mixture of multiple and single choice, open formatted questions, and calculations. The difficulty level of the items ensures that the exams also require solving problems based on knowledge application. This is important to assume grades as a proxy for Gc. Thus, performance in the exams can be regarded as an indicator of Gc. The internal consistency for this GPA was *α* = 0.78.

### 2.4. Statistical Procedure

**Preliminary analyses and variance decomposition.** First, zero-order correlations were calculated to explore covariation on the between-person level. In order to estimate the intraindividual variability of Openness and interests, intercept only multilevel models were fit for each variable and intraclass correlation coefficients (ICC) calculated as a measure of the ratio of variance between persons to the total variance [55]. This is a test of Hypothesis 1.

**Within-person process.** Next, in order to test whether there is within-situation covariation between Openness and interests, within- and between-person relationships were examined via multiple steps of multilevel modeling, using linear mixed effects regression (LMER) from the package *lme4* version 1.0-5 [56] in R version 3.0.2 [57]. This is a test of Hypothesis 2. Moreover the package *apaTables* [58] was used.

**Multilevel modeling.** In order to test whether the assumed intraindividual covariation was systematic, following the typical step-up procedure a multilevel model was built from bottom up (through nested models “0”, “1” and “2”), first separately for the four interest variables: psychology, investigative, artistic, and social interest (“A” to “D”) to determine whether each covaried with Openness. Here we expected covariance mainly for investigative and psychology interest. Following these analyses, a combined model including all interests was tested (“E”, controlling for overlap) to determine whether and to what extent the covariations are specific. First, for the null model (“0”), a model of state Openness as the dependent variable, with random intercepts and without correlates, was fit (allowing for intercept variation reflects that different participants may have different average Openness scores). For the random intercept models (“A1” to “E1”) interest in psychology or/and investigative, artistic, and social interest were then entered as fixed effect predictors—assuming covariation of interests with Openness common to all participants. Finally, for the random coefficients models (“A2” to “E2”) random effects for slopes of Openness on the respective interests were entered—allowing for individual covariations between interests and Openness. Full Maximum-likelihood estimation was used for all LMER models. Subsequent model comparisons were realized via likelihood-ratio-tests. To quantify the variance explained through added variables the reduction of variance on levels 1 and 2 was calculated according to [59].

**Transformation of predictor variables.** Level 1 presents the variation of self-ratings across measurement occasions within persons and level 2 refers to the variation between persons. Openness and interests were assumed to vary both over time *and* on average. To disentangle within-person from between-person effects, the predictor variables at level 1 (interests) were centered at the person’s own mean scores across days (person-mean centering; see [60,61]). For the aforementioned models, both components of each predictor (person means at level 2 and deviations from the corresponding means at level 1) were entered simultaneously to produce correct estimates of effects at both levels.

**Effects on accumulation of knowledge.** Finally, in order to explain the development of knowledge as a result of the interaction between an indicator of Gf and the covariation between Openness and interest (as a measure of willingness to engage), a blockwise multiple linear regression was conducted. More specifically, the individual slopes for the covariation between Openness and interest indicators were extracted from the multilevel models with only one interest as correlate (“A2” to “D2”). Considering the small sample size we only considered those relations, which rendered a significant fixed effect in the combined multilevel model (“E2”). These individual coefficients were used as predictors in a multiple regression with GPA as dependent variable. Moreover, test scores for the corresponding traits as well as for Openness and Gf were entered in Block 1. The Gf test score also served as moderator variable for both slopes in Block 2. Hypothesis 3 would be confirmed if the interaction between the indicator of Gf and the individual covariation between Openness and interest were significant.

### 2.5. Power Considerations

Estimating power for multilevel models is not a straightforward procedure. Most analyses focus on the covariation between states on level 1. However, testing Hypothesis 3 also includes level 2. According to Scherbaum and Ferreter [62] each of these effects requires different power considerations. Maas and Hox [63] recommended using at least 50 level 2 units to obtain sufficient power and adequate standard errors. However, the literature is less clear on recommendations regarding power to detect cross-level interactions. Van der Leeden and Busing [64] recommended using 30 units on each level. Yet, they also stated that fewer level 1 units are reasonable with more level 2 units. Scherbaum and Ferreter [62] provide a ready to use R code to estimate power or optimal sample size. However, the code needs parameter estimates from a prior or pilot study. Unfortunately, no such pilot study exists. We therefore, decided to follow the rule of thumb of 50 level 2 units. We hope that the found results can serve as priors for power analyses of ensuing research.

More information regarding all measures used and the R code can be found under the OSF link: https://osf.io/4u7br/?view_only=334e6d2ab82f47e5a9db87e49d3f5393.

## 3. Results

### 3.1. Preliminary Analyses and Variance Decomposition

**Between-person covariation.** Descriptive statistics, ICCs, and zero-order correlations can be found in Table 1. Correlations between Openness and interest in psychology, as well as Investigative, Artistic, and social interests were positive and significant.

**Within-person variation.** As shown by the ICCs in Table 1 the ratio of total variance across time explained by differences between persons was 28% for the Openness indicator, 30% for the interest in psychology indicator, 40% for the investigative interest indicator, 43% for the artistic interest indicator, and 49% for the social interest indicator. These relatively small proportions of variance at the person-level indicate that the measures strongly vary across days. These findings support Hypothesis 1.

**Figure 3:**
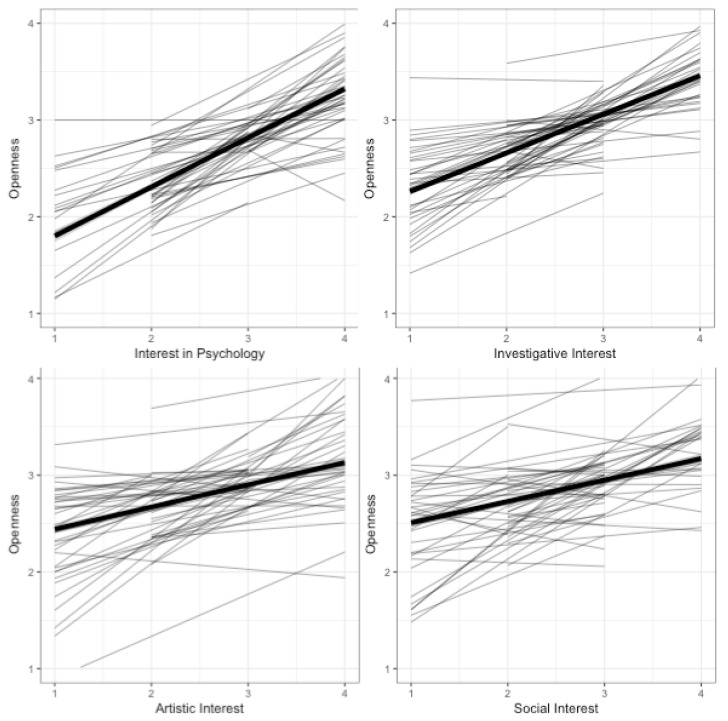
Regression lines for Openness as a function of interests, person-specific (thin) and average (thick).

**Within-person covariation**—**descriptively.**Figure 3 presents fitted regression lines for each participant (thin) and the average (thick) for the Openness indicator as a function of the interest indicators. Visual inspection suggested substantial positive relations on the between as well as the within-level between the Openness indicator and the interest in psychology and investigative interest indicators (with some between-person variability in intercepts and slopes), and smaller in size between the Openness and artistic and social interests indicators. This can be seen as support for Hypothesis 2.

**Within-person covariation**—**model testing.** Fit information for the hypothesized models using the Openness indicator as dependent variable and relevant comparisons are shown in Table 2. The models are arranged according to the planned step-up procedure (at the same time, pairwise comparisons are sorted in terms of goodness of fit, with better-fitting models in subsequent rows). As the table shows, successive comparisons of the nested models yield random coefficients models (“A2” to “E2”) fitting best in all cases. Finally, the combined interests model (“E2”) could be shown to fit better than each of the best-fitting models with only one predictor.

The fixed between effects were significant in all models containing only one interest indicator as correlate (see Table 3). In the combined model, only the effects for the indicators of psychological and investigative interests remained significant. This means that there was a positive overall covariation between the indicator for Openness and indicators of psychological and investigative interest. On the within-person level, there were significant covariations in all cases. However, the covariation was stronger for interest in psychology and investigative interest. As mentioned above, model E had the best fit and will be further inspected. A total of 47% of differences in Openness between persons (*R*^2^ level 2) and 35% of differences within persons (*R*^2^ level 1) was related to the four interest variables. In line with our hypothesis average relations of Openness with interests were positive for all interest variables on the within-level. To get an impression of the range of the correlations, we calculated plausible value ranges of within-person relations [59]. Noticeably, broad plausible value ranges were found: 95% of the correlations for Openness and interest in psychology fell within a range of −0.18 to 0.75, −0.15 to 0.52 for Openness and investigative interest, −0.51 to 0.75 for Openness and artistic interest, and −0.38 to 0.53 for Openness and social interest. This indicates the presence of moderator variables.

### 3.2. Effects on Accumulation of Knowledge

The third hypothesis to be tested was that the interaction between willingness to engage and an indicator of Gf positively affected the acquisition of knowledge as measured by the grades. To test the hypothesis, estimates of these within-person coefficients were used to predict GPA in psychology while controlling for scores from the Gf, Openness, and interest tests, measured at the lab session. The Gf test score also served as moderator in a second block. Considering the non-significant fixed effects for social and artistic interests within the last analyses, we conducted this analysis for the indicators of investigative interest and interest in psychology only. The results can be found in Table 4.

It can be seen that adding the interaction terms increased the amount of explained variance by eight percent. Yet, this was not significant using the current sample size. With regard to the regression weights, there were only two marginally significant effects: Higher scores in the Openness questionnaire improved performance as did the interaction between the Gf indicator and the within-person covariation between interest in psychology and the Openness indicator. Of interest is the interaction which means that larger Gf scores make the impact of the within-person covariation between interest in psychology and Openness on grades more negative and thereby improve performance. At the same time, the two covariations themselves, which reflect willingness to engage with new stimuli, did not affect exam performance.

## 4. Discussion

The main aim of this paper was to propose a substantial elaboration of the OFCI model. Additionally, results from a first test, meant as a pilot study, for some of the ideas are presented. In particular, we tested whether an indicator of Intellect and thus Openness varies across situations, which could be confirmed. Moreover, it could be shown that this variation was systematically linked to variation in specific interests, especially investigative and domain-specific interest. Finally, we found descriptive support for the idea that this systematic covariation interacts with an indicator of Gf influencing academic achievement as an indicator of knowledge. While these results are certainly encouraging, they also highlight some of the problems future research needs to tackle. For example, there were strong interindividual differences in covariation between Openness and interest states. We assume that differences in situational perceptions can account for some of this variance.

### 4.1. (Co-)Variation of Openness and Interests

The current results support the notion of situational fluctuation within Openness. The current findings are similar to previous studies. For example, Wilson, Thompson, and Vazire [40] reported that more than 60% of the total variance of Openness in an experience sampling was due to within-person fluctuations. Interestingly, Finnigan and Vazire [66] advised to first refrain from measuring Openness as a state, assuming its nature as captured in items to be too trait-like. The same authors later corrected this and proceeded with measuring Openness states resulting in substantial amounts of fluctuation similar to the ones found here.

As mentioned above, with regard to interests, there is little research testing situational fluctuation of interests. Tsai et al. [67] reported fluctuations for domain specific interests for students in school in the subjects math, German, and foreign language. They estimated the amount of total variance due to within-person variation to be between 36% and 45%. Knogler, Harackiewicz, Gegenfurtner and Lewalter [41] scrutinized the experience of momentary, so-called *situational* interest in repeated instructional activities. Applying latent state-trait modeling, the authors described substantial variance, about 50%, to be situation-specific. These results are line with our findings further supporting the informative value of the current data. Thus, on the whole, Hypothesis 1 was supported.

Hypothesis 2 stated that there would be substantial within-person covariation between Openness and interests. The current findings support this assumption. While there were suggestive fixed effects, implying a positive covariation between Openness and interests, specifically interest in psychology and investigative interest, there also were substantial interindividual differences. The estimated plausible values range from small negative values to moderate to large positive values. Thus, for some people the assumed covariation across situations is rather strong while it is virtually absent for others. This implies the existence of moderators. The most straightforward explanation could be differences in situations encountered. However, data were collected within a student cohort during the semester. Thus, at least for weekdays the schedule should have been very comparable until the early evening hours, making situations somewhat comparable.

Other potential moderators could be situational perceptions. So far we have treated situational perceptions as states only. Ziegler, Horstmann, and Ziegler [68] (conditionally accepted) provide evidence for the existence of trait-like components. In other words, some people might tend to perceive situations as for example Intellect-laden resulting in Openness and interest manifestations. Others might tend to perceive situations as psychologically stressful, preventing such manifestations. To overcome such general blockers, the situational cues related to situational perceptions facilitating the manifestation of Openness and interests would have to be very strong. Thus, in order to explore the role of situational perceptions for environmental enrichment future research should include state and trait measures of situational perception to test their moderating role on the relation between Openness and interest.

Finally, it could also be that the individual profile of Openness and interests moderates their correlation. For example, people high on the Seek but low on the Conquer facet of Intellect might not establish lasting behavioral patterns being easily bored by the repetition of initially new and interesting situations. This would impede an enduring covariation between Openness and interest. There might be further motivational variables impacting the manifestation of Openness and interests and thereby affecting their covariation. Zhang and Ziegler [69] postulated and successfully tested the idea that the Big Five exert their influence on learning through the activation of self-beliefs and learning strategies. Those authors could show that self-concept and a deep-learning approach mediated the influence of Openness on learning. All of these ideas bear implications for future research. It seems reasonable to include more differentiated measures covering the whole range of constructs used here as explanatory variables. Furthermore, the role of additional constructs important for learning such as self-concept and learning strategies needs to be explored.

### 4.2. The Influence on Gc

Based on a multiple regression we tested several of the ideas postulated above. Unfortunately, the power of this study to test this final model was too low. Thus, no significant results emerged. Based on the effects found, a sample of more than 120 would be needed to have sufficient power to test the interaction effects in a regression model^1^. Nevertheless, we did find some effects deserving a closer examination. For what it might be worth, those effects show that Openness was predictive of better grades. This was also true for investigative interests. However, here the effect direction was reversed. It can be assumed that the criterion used here, written exams, required a lot of rote learning. A strong investigative interest might take too much time away from rote learning, maybe invested in reading additional literature, yielding such a negative effect. Positive effects might occur after a longer time period. In that sense, the current criterion can be considered to be contaminated [70,71].

The assumed interaction between the intraindividual covariation of Openness and interest and Gf predicting learning was also not statistically significant. Again, only a marginally significant result occurred and only for the interaction between interest in psychology and Gf. Several lessons can be learned here, using larger samples being the most obvious one. Additionally, it seems advisable to use broader measures of Gf, including, for example, verbal and numerical tests as well. Specifically, verbal reasoning has been shown to be predictive of success in psychology [20]. Finally, the fact that our domain-specific measure yielded the best result also implies that the use of generally phrased interest measures might be too unspecific. Thus, future studies should continue to use domain specific interest measures.

### 4.3. Limitations

The empirical study comes along with a number of limitations. Using a small set of variables without explicit temporal order on the micro-level imposed limits on resolving the causal relation between states of Openness and interests. Due to the multitude of constructs operationalized in the experience sampling each was assessed by only one item. Perhaps, the use of only one item per construct is the most problematic limitation. Clearly, the measures within situations are less reliable and also less construct valid. Thus, the current results can only be regarded as lower bound estimates. Future studies need to utilize more items to overcome this limitation. Related, the items measuring Openness and investigative interests might appear to be too close with regard to content. Yet, as the results show, there is variability with regard to the correlation between those two items. Moreover, such a content overlap can be ruled out for the item assessing interest in psychology. Still, the results found for the investigative item also occurred for this item.

The specific sample used could also entail a restriction of range in some of the constructs focused here. Psychology students in Germany are mostly selected based on their high school GPA. It is reasonable to assume that the GPA is influenced by Gf and personality [72]. As mentioned above, psychology students usually also have specific interest profiles [29]. Thus, range restrictions for Openness, interests, and Gf are at least plausible. This would also yield smaller effects, again rendering lower bound estimates. It has to be noted though that the use of a specific sample also had many advantages. First, selecting the interest measures was easier exactly due to the assumed general preferences. Moreover, some standardization of situations could be assumed. Thus, future studies need to find a balance between sample nature and potential range restrictions.

As a highly unique sample was analyzed here, consisting of university students of only one faculty, interest items specific to this field of study, and first-semester exams as an outcome measure, of course, the generalizability of the results needs to be determined. Future studies may replicate our findings in different contexts, with measures of interests and outcomes covering diverse areas of knowledge. It would also be interesting to apply experimental designs, systematically varying the amount of situational cues potentially activating investigative interests.

Another limitation is the lack of assessment of Gf on a daily basis. Thus, the processes by which Openness and interests influence the accumulation of knowledge suggested in the elaboration of the OFCI model cannot be comprehensively tested. However, it can safely be assumed that variations in Gf might be much smaller than in the other traits. Danner, et al. [73] could show that measures of intelligence are virtually unaffected by state variance.

Schmidt [74] published a model of academic and occupational achievement in which he linked introversion to the acquisition of specialized knowledge. Not controlling for introversion might lead to an overestimation of the openness effect found here. However, as our focus was on the openness-interest link, this might have little overall consequence. Mount, Barrick, Scullen, and Rounds [27] reported in their meta-analysis a true correlation of 0.02 between extraversion and investigative interest. Moreover, there is no strong empirical support for a longitudinal relation between extraversion and cognitive abilities [75].

Finally, we did not utilize a pure Gc measure. Instead, we used grades achieved over the course of several semesters. Such grades reflect mastery of specialized knowledge in psychology. As such, the grades are indicators of domain specific knowledge (Gkn), which is an integral part of all acquired knowledge, and can conceptually be situated as a branch of verbal knowledge (Gc) according to Schneider and McGrew ([76], see Figure 4.9). Those authors (p. 122) also noted that the CHC components of Gc, Gkn, Grw (general knowledge of reading and writing), and Gq (quantitative knowledge) are consistent with Cattell’s notion of Gc [77]. Thus, with regard to terminology, we are caught between a rock and a hard place. Using grades should therefore be seen as a way of operationalizing knowledge. In CHC terms, the grades used here reflect specialized knowledge. Future studies should try to utilize other operationalizations. Yet, two aspects need to be considered when trying to test the OFCI model’s predictions. First, a pure measure of Gc in the CHC sense might be illusory. Most tests will focus on one or the other facet. Moreover, the OFCI model as presented here does not propose an increase in all domains of Gc. Instead it is assumed that the increase will be influenced by interests. Thus, using a sample with homogenous interests is advantageous. At the same time this will also most likely mean that a specialized knowledge test is warranted. Second, large parts of Gc according to the CHC model are aspects like reading, writing, or listening ability. It can safely be assumed that, from a certain age on, variance in these aspects can be restricted. Thus, very difficult tests are needed. Alternatively, very young participants might have to be included. However, for the latter the OFCI model would predict that the strong degree of standardization and, in a sense, the strong situation would prevent differences in Openness to manifest [15,16]. Consequently, integrating a pure Gc measure into any study is a challenge. We here propose to accumulate knowledge by conducting studies in different samples, focusing on different Gc aspects while scrutinizing situational demands and test difficulty.

## 5. Conclusions

The current paper introduces an important theoretical elaboration of the OFCI model. Specifically, the within-situation interplay between Openness, interests, situational perceptions, emotions, and abilities was elaborated, and selected parts of this elaboration empirically tested. This is an important step in further shedding light onto the intricate interplay and development of personality and ability. Despite the obvious limitations of the empirical study, there is first evidence supporting the ideas brought forward. Future studies can use the parameters estimated here to conduct more substantial power analyses yielding superior designs. Nevertheless, the current paper shows that the process of learning and the development of Openness, interests, and abilities are closely intertwined. Additionally, this paper also shows the necessity to integrate research within and across situations to establish and test mechanisms of learning and development.

## Figures and Tables

**Table 1:** Means, standard deviations, and correlations with confidence intervals.

Variable	*M*	*SD* (*ICC*)	1	2	3	4	5	6	7	8	9	10	11
1. Gf	0.61	0.21											
2. Grades	2.92	0.65	0.19										
			[−0.10, 0.44]										
3. Openness	3.87	0.33	−0.14	0.10									
			[−0.39, 0.13]	[−0.18, 0.37]									
4. Interest in Psychology	4.01	0.37	−0.06	0.02	0.38								
			[−0.32, 0.21]	[−0.26, 0.29]	[0.13, 0.59]								
5. Investigative Interest	3.03	0.70	−0.10	−0.15	0.46	0.54							
			[−0.36, 0.18]	[−0.41, 0.13]	[0.21, 0.65]	[0.32, 0.71]							
6. Artistic Interest	3.77	0.83	−0.22	0.12	0.68	0.21	0.28						
			[−0.46, 0.06]	[−0.17, 0.38]	[0.51, 0.81]	[−0.06, 0.46]	[0.01, 0.51]						
7. Social Interest	3.85	0.61	−0.00	0.04	0.39	0.28	0.29	0.36					
			[−0.27, 0.27]	[−0.24, 0.32]	[0.13, 0.59]	[0.01, 0.51]	[0.02, 0.52]	[0.09, 0.57]					
8. Openness Mean State	2.81	0.35 (0.28)	−0.05	0.21	0.44	0.20	0.29	0.30	0.13				
			[−0.32, 0.22]	[−0.07, 0.46]	[0.19, 0.63]	[−0.07, 0.45]	[0.02, 0.52]	[0.03, 0.53]	[−0.15, 0.39]				
9. Interest in Psychology Mean State	2.98	0.35 (0.30)	−0.08	0.11	0.29	0.20	0.19	0.11	0.27	0.61			
			[−0.34, 0.19]	[−0.17, 0.37]	[0.03, 0.52]	[−0.07, 0.44]	[−0.08, 0.44]	[−0.16, 0.37]	[0.00, 0.51]	[0.41, 0.75]			
10. Investigative Interest Mean State	2.33	0.52 (0.40)	−0.03	0.20	0.38	0.32	0.44	0.40	0.16	0.72	0.49		
			[−0.30, 0.24]	[−0.08, 0.45]	[0.13, 0.59]	[0.05, 0.54]	[0.20, 0.64]	[0.14, 0.60]	[−0.11, 0.42]	[0.55, 0.83]	[0.26, 0.67]		
11. Artistic Interest Mean State	2.55	0.56 (0.43)	−0.16	0.21	0.23	0.10	0.13	0.51	0.22	0.29	0.23	0.53	
			[−0.41, 0.11]	[−0.07, 0.46]	[−0.04, 0.47]	[−0.18, 0.35]	[−0.15, 0.38]	[0.27, 0.68]	[−0.05, 0.47]	[0.03, 0.52]	[−0.04, 0.47]	[0.30, 0.70]	
12. Social Interest Mean State	2.31	0.58 (0.48)	0.04	0.29	0.14	0.20	0.01	0.30	0.34	0.36	0.33	0.49	0.34
			[−0.23, 0.30]	[0.02, 0.52]	[−0.13, 0.39]	[−0.07, 0.45]	[−0.26, 0.28]	[0.04, 0.53]	[0.08, 0.56]	[0.10, 0.57]	[0.07, 0.55]	[0.26, 0.67]	[0.08, 0.56]

*Note*. Sample size varied from 50 to 54 due to missing data. *ICC* = intraclass correlation coefficient. Shown for ESM measures only. Gf = ratio of correct solutions in RAPM as indicator of fluid intelligence (range: 0–1), Grades = GPA of grades in psychology as indicator of Gkn (range: 1–4 with higher values indicating better performance), Openness (range: 1–5), Interest in psychology, Investigative interest, Artistic interest, Social interest ranges: 1–5). *M* and *SD* are used to represent mean and standard deviation, respectively. Values in square brackets indicate the 95% confidence interval for each correlation. The confidence interval is a plausible range of population correlations that could have caused the sample correlation [65].

**Table 2:** Fit information for hypothesized models predicting openness and relevant comparisons.

	Model	*AIC*	*p*
0	Null model (random intercepts, no predictors)	5568.7	
A1	Random intercept model with interest in psychology as predictor	4842.3	<0.001 ^a^
A2	Random coefficients model with interest in psychology as predictor	4765.1	<0.001
B1	Random intercept model with investigative interest as predictor	5071.9	<0.001 ^a^
B2	Random coefficients model with investigative interest as predictor	4973.2	<0.001
C1	Random intercept model with artistic interest as predictor	5291.6	<0.001 ^a^
C2	Random coefficients model with artistic interest as predictor	5186.9	<0.001
D1	Random intercept model with social interest as predictor	5389.4	<0.001 ^a^
D2	Random coefficients model with social interest as predictor	5291.4	<0.001
E1	Random intercept model with all four interests as predictors	4456.1	<0.001 ^a^
E2	Random coefficients model with all four interests as predictors	4413.1	<0.001 ^b^

*Note*. *AIC* = Akaike information criterion. ^a^ Compared to null model (“0”). ^b^ Compared to all random intercept models (“A1” to “E1”).

**Table 3:** Parameter estimates for multilevel models of openness as a function of interests.

Model	A		B		C		D		E	
1: Fixed Effects	Estimate	*t*	Estimate	*t*	Estimate	*t*	Estimate	*t*	Estimate	*t*
Psychology Interest (within)	0.41 (0.32)	12.45							0.29 (0.03)	9.30
Psychology Interest (between)	0.60 (0.11)	5.63							0.32 (0.09)	3.56
Investigative Interest (within)			0.32 (0.03)	10.77					0.19 (0.02)	8.67
Investigative Interest (between)			0.52 (0.06)	8.79					0.48 (0.07)	6.67
Artistic Interest (within)					0.26 (0.03)	8.38			0.13 (0.02)	6.20
Artistic Interest (between)					0.19 (0.08)	2.35			−0.05 (0.05)	−0.8
Social Interest (within)							0.23 (0.03)	6.75	0.07 (0.02)	2.92
Social Interest (between)							0.23 (0.08)	3.03	−0.05 (0.05)	−0.93
2: Random Effects (variances)	Estimate	*SD*	Estimate	*SD*	Estimate	*SD*	Estimate	*SD*	Estimate	*SD*
Psychology Interest (within)	0.04	0.20							0.03	0.17
Investigative Interest (within)			0.03	0.19					0.01	0.11
Artistic Interest (within)					0.04	0.19			0.01	0.10
Social Interest (within)							0.04	0.21	0.01	0.11

**Table 4:** Regression results using grades as the criterion.

Predictor	*b*	*b* 95% CI [*LL*, *UL*]	*beta*	*beta* 95% CI [*LL*, *UL*]	*sr* ^2^	*sr*^2^ 95% CI [*LL*, *UL*]	*r*	Fit	Difference
Block 1									
(Intercept)	2.68 **	[2.04, 3.32]							
Raven	0.14	[−0.05, 0.32]	0.22	[−0.07, 0.51]	0.05	[−0.06, 0.15]	0.19		
Openness	0.18	[−0.04, 0.40]	0.27	[−0.06, 0.60]	0.06	[−0.06, 0.17]	0.13		
Interest in Psychology	0.06	[−0.17, 0.30]	0.10	[−0.26, 0.46]	0.01	[−0.03, 0.05]	0.04		
Investigative Interest	−0.22	[−0.46, 0.03]	−0.33	[−0.71, 0.05]	0.06	[−0.06, 0.19]	−0.15		
Within 1: Openness × Interest in Psychology	0.71	[−0.66, 2.07]	0.18	[−0.17, 0.52]	0.02	[−0.05, 0.10]	0.07		
Within 2: Openness × Investigative Interest	−0.26	[−1.72, 1.21]	−0.06	[−0.42, 0.30]	<0.01	[−0.02, 0.03]	−0.05		
								*R*^2^ = 0.145	
								95% CI [0.00, 0.24]	
Block 2									
(Intercept)	2.79 **	[2.14, 3.44]					2.79 **		
Raven	0.10	[−0.08, 0.28]	0.16	[−0.13, 0.45]	0.02	[−0.05, 0.10]	0.10		
Openness	0.20	[−0.02, 0.41]	0.29	[−0.03, 0.61]	0.06	[−0.06, 0.18]	0.20		
Interest in Psychology	0.11	[−0.14, 0.35]	0.16	[−0.21, 0.54]	0.01	[−0.04, 0.07]	0.11		
Investigative Interest	−0.21	[−0.45, 0.04]	−0.32	[−0.70, 0.05]	0.06	[−0.06, 0.17]	−0.21		
Within 1: Openness × Interest in Psychology	0.81	[−0.57, 2.20]	0.20	[−0.14, 0.55]	0.03	[−0.05, 0.11]	0.81		
Within 2: Openness × Investigative Interest	−0.71	[−2.21, 0.78]	−0.18	[−0.54, 0.19]	0.02	[−0.05, 0.08]	−0.71		
Within 1 × Gf	0.22	[−0.01, 0.45]	0.32	[−0.02, 0.66]	0.07	[−0.06, 0.19]	0.22		
Within 2 × Gf	−0.14	[−0.35, 0.06]	−0.22	[−0.54, 0.10]	0.04	[−0.06, 0.13]	−0.14		
								*R*^2^ = 0.226	Δ*R*^2^ = 0.08
								95% CI [<0.01, 0.31]	95% CI [−0.05, 0.21]

*Note*. * indicates *p* < 0.05; ** indicates *p* < 0.01. A significant *b*-weight indicates the beta-weight and semi-partial correlation are also significant. *b* represents unstandardized regression weights; *beta* indicates the standardized regression weights; *sr^2^* represents the semi-partial correlation squared; *r* represents the zero-order correlation. *LL* and *UL* indicate the lower and upper limits of a confidence interval, respectively. Higher grades indicate better performance.

## Footnotes

1 The material on the OSF page also includes Table i showing regression analyses for a reduced set of predictors as recommended by an anonymous reviewer.
